# Regulation of Cadmium-Induced Proteomic and Metabolic Changes by 5-Aminolevulinic Acid in Leaves of *Brassica napus* L.

**DOI:** 10.1371/journal.pone.0123328

**Published:** 2015-04-24

**Authors:** Basharat Ali, Rafaqat A. Gill, Su Yang, Muhammad B. Gill, Muhammad A. Farooq, Dan Liu, Muhammad K. Daud, Shafaqat Ali, Weijun Zhou

**Affiliations:** 1 Institute of Crop Science and Zhejiang Key Laboratory of Crop Germplasm, Zhejiang University, Hangzhou 310058, China; 2 Department of Biotechnology and Genetic Engineering, Kohat University of Science and Technology, Kohat 26000, Pakistan; 3 Department of Environmental Sciences and Engineering, Government College University, Faisalabad 38000, Pakistan; Universidade Federal de Vicosa, BRAZIL

## Abstract

It is evident from previous reports that 5-aminolevulinic acid (ALA), like other known plant growth regulators, is effective in countering the injurious effects of heavy metal-stress in oilseed rape (*Brassica napus* L.). The present study was carried out to explore the capability of ALA to improve cadmium (Cd^2+^) tolerance in *B*. *napus* through physiological, molecular, and proteomic analytical approaches. Results showed that application of ALA helped the plants to adjust Cd^2+^-induced metabolic and photosynthetic fluorescence changes in the leaves of *B*. *napus* under Cd^2+^ stress. The data revealed that ALA treatment enhanced the gene expressions of antioxidant enzyme activities substantially and could increase the expression to a certain degree under Cd^2+^ stress conditions. In the present study, 34 protein spots were identified that differentially regulated due to Cd^2+^ and/or ALA treatments. Among them, 18 proteins were significantly regulated by ALA, including the proteins associated with stress related, carbohydrate metabolism, catalysis, dehydration of damaged protein, CO2 assimilation/photosynthesis and protein synthesis/regulation. From these 18 ALA-regulated proteins, 12 proteins were significantly down-regulated and 6 proteins were up-regulated. Interestingly, it was observed that ALA-induced the up-regulation of dihydrolipoyl dehydrogenase, light harvesting complex photo-system II subunit 6 and 30S ribosomal proteins in the presence of Cd^2+^ stress. In addition, it was also observed that ALA-induced the down-regulation in thioredoxin-like protein, 2, 3-bisphosphoglycerate, proteasome and thiamine thiazole synthase proteins under Cd^2+^ stress. Taken together, the present study sheds light on molecular mechanisms involved in ALA-induced Cd^2+^ tolerance in *B*. *napus* leaves and suggests a more active involvement of ALA in plant physiological processes than previously proposed.

## Introduction

Heavy metals such as cadmium, lead and chromium are almost persistent environmental pollutants. Among them, cadmium (Cd^2+^) is highly toxic [1], and has greater hazardous impact on ecosystem. Its different sources are pesticides and chemical fertilizers, which easily release Cd^2+^ into our environment. It belongs to a group of metals, which are water soluble [2, 3]. Among the well-known phyto-toxic heavy metals in the environment, Cd^2+^ is of considerable importance due to its high water solubility, mobility, persistence, and toxicity even in minute amounts [4]. Using roots as medium of entry, Cd^2+^ enters our food chains and causes serious human health problem such as cancer.

The oilseed rape (*Brassica napus* L.) is a worldwide source of edible oil [5]. *Brassica* species are generally considered as tolerant to heavy metal, due to their fast growth, more biomass and ability to absorb heavy metals [6]. *Brassica* plants adopt different strategies to overcome or tolerate metal toxicity by specific mechanisms in heavy metal contaminated soils [7].

The toxicity effects of Cd^2+^ in plants have been extensively studied, although several questions remain unaddressed [8]. Symptoms of Cd^2+^ toxicity in plants include growth inhibition and the disruption of physiological processes [9]. Plants have evolved mechanisms for alleviating and/or tolerating heavy metal stress, including Cd^2+^ stress, some of which include: exclusion, compartmentalization, complexation/sequestration by small molecules such as phytochelatins (PCs), and the synthesis of stress-response proteins [4]. Cd^2+^ causes several toxicity symptoms in plants at various functional levels such as physiological, biochemical and proteomic. Physiological disturbances such as water transport, absorption and transport of essential elements, oxidative phosphorylation in mitochondria, photosynthesis, respiration, chlorophyll content, plant growth and reproduction in plants have been reported [10]. Cd^2+^ also disturbs different metabolic processes and can cause decline of water and nutrient uptake in plants [11]. Plants contain antioxidative mechanisms to avoid the oxidative stress such as superoxide dismutase (SOD), catalase (CAT) and peroxidase (POD) as well as glutathione, carotenoids and ascorbate [12]. In plants, SOD dismutase’s the O_2_^-^ to H_2_O_2_, and CAT scavenges the H_2_O_2_, which is generated from photorespiration [13]. POD utilizes H_2_O_2_ in the oxidation of different organic and inorganic substrates and it is located in vacuole, cell wall as well as in extracellular spaces [14].

The plant growth regulators (PGRs) are often used to increase the stress tolerance of plants. Similarly, 5-aminolevulinic acid (ALA) is one of most important plant growth regulator, which is known as essential precursor for the biosynthesis of tetrapyrrols like protochlorophyllide (that is converted in chlorophyll when exposed to light) [15, 16]. In previous research, we found that ALA alleviates the Cd^2+^ toxicity by improving the plant growth and ultrastructural changes in oilseed rape [16]. Moreover, Wang et al. [17] also showed that treatment of ALA exercised a positive effect on the growth of *Brassica campestris* seedlings. Recently, it was observed that ALA improved the osmotic potential and relative water content in *B*. *napus* under salinity stress. Moreover, it was also found that soluble sugar and free amino acid contents were enhanced with the application of exogenous ALA in *B*. *napus* under salinity stress [18].

It is evident from a number of previous reports that ALA, like other known PGRs, is effective in countering the injurious effects of various abiotic stresses on plants, but the information regarding the molecular mechanisms about the question how ALA regulates plant growth under stressful situation are not fully elucidated yet. Proteomic study is achieving great recognition as a reliable and reproducible high-through put approach in understanding biological processes under various environments [19]. It is based on the systematic analysis and documentation of expressed proteins and their study at the functional level [20]. The present experiment is designed to study the comprehensive knowledge about the mechanisms how ALA regulates the physiological, metabolic and proteomic changes in leaves of *B*. *napus* under Cd^2+^ stress.

## Materials and Methods

### Plant material and growth conditions

For the experiment, seeds of winter *Brassica napus* L. cv. ZS 758 were obtained from the College of Agriculture and Biotechnology, Zhejiang University. The seeds were grown in plastic pots (170 mm×220 mm) filled with peat moss. At five leaf stage, morphologically uniform seedlings were selected and plugged these seedlings into plate holes on plastic pots (six plants per pot) containing a half strength Hoagland nutrient solution [21], aerated continuously with an air pump, in the greenhouse. The composition of Hoagland nutrient solution was as follows (in μmol/L): 3000 KNO_3_, 2000 Ca(NO_3_)_2_, 1000 MgSO_4_, 10 KH_2_PO_4_, 12 FeC_6_H_6_O_7_, 500 H_3_BO_3_, 800 ZnSO_4_, 50 Mncl_2_, 300 CuSO_4_, 100 Na_2_MoO_4_. The pH of solution was maintained at 6.0. The light intensity was in the range of 250–350 μmol m^-2^ s^-1^, temperature was16–20°C and the relative humidity was approximately 55–60%. Each treatment was replicated four times. The nutrient solution was renewed every 5 days.

After acclimatization period of seven days, Cd^2+^ as CdCl_2_ was added into full-strength Hoagland solution making desired Cd^2+^ concentrations (0, 100 and 500 μM) and plants were simultaneously sprayed with or without an aqueous solution of ALA (Cosmo Oil Co. Ltd., Japan) at a concentration of 25 mg/L ALA. The treatment concentrations were based on pre-experimental studies, in which several lower and higher levels of metal were used, i.e., 100, 200, 300, 400, 500 and 1000 μM of CdCl_2_. The Cd^2+^ at 100 μM showed a little damage on plant growth and 500 μM Cd^2+^ imposed a significant damage to plant growth, while those higher than 500 μM were too toxic for plant growth. It was similar in the case of ALA application, where plants exhibited optimum response with the treatment of 25 mg/L concentration under Cd^2+^ stress conditions [16, 22]. The lower, as well as the upper leaf surfaces were sprayed until wetted with a hand-held atomizer, as it was reported that absorption by the lower leaf surface is rapid and effective [23]. After the five days of first spray, subsequent application was followed. Plants sprayed with distilled water served as the control. Fifteen days after treatment, samples for biochemical, metabolic and proteomic studies of leaf were collected as described below.

### Osmotic potential and relative water content

The leaves for the measurement of leaf osmotic potential (*ψ*_*s*_) were frozen in liquid nitrogen and stored at -80°C. The samples were thawed for 30s, which provided 10 μL of sap that was used for the determination of osmotic potential according to Gucci et al. [24] using a vapor pressure osmometer (Wescor Inc., Logan, UT, USA).

The relative water contents (RWC) were determined in fresh leaves, excluding midrib. Samples were weighed quickly and immediately floated on double distilled water, in Petri dishes to saturate them with water for the next 24 hr, in dark. The excessive water was blotted and turgor weight was taken. Dry weight of these samples was obtained, after dehydrating them at 70°C for 48 hr. RWC were calculated by placing the observed values in the following formula:

$$\text{RWC }=\;\frac{\text{Fresh weight}–\text{Dry weight}}{\text{Turgid weight}–\text{Dry weight}}\;\times 100$$

### Soluble sugar, free amino acid and proline contents

The soluble sugars contents were estimated as reported by Zhang et al. [15]. Fresh leaves were boiled in distilled water in water bath for 30 min and then centrifuged at 2,000 g for 15 min. The supernatants were used for the sugar analysis by using spectrophotometric method. After that, soluble sugar concentration was determined. Total free amino acids were estimated according to the method of Yemm and Cocking [25]. One mL of amino-acid solution was mixed with 2 mL sodium acetate buffer (pH 6.5) and 1 mL freshly prepared ninhydrin reagent. The resulting color was determined at 570 nm.

The proline contents in fresh leaves were determined by adopting the method of Bates et al. [26]. Samples were extracted with sulfo-salicylic acid. In the extract, an equal volume of glacial acetic acid and ninhydrin (1.25 g ninhydrin, 30 mL of glacial acetic acid, 20 mL of 6 M H_3_PO_4_) solutions were added. The samples were incubated at 100°C for 40 min, to which 3 mL of toluene was added. The absorbance of the toluene layer was read at 520 nm, on a spectrophotometer. After that, proline concentration was determined.

### Measurements of chlorophyll fluorescence parameters

Photochemical quenching parameters (F0, Fm, Fm´ and Fv/Fm) were measured using an imaging pulse amplitude-modulated (PAM) fluorimeter (IMAG-MAXI; Heinz Walz, Effeltrich, Germany). The F0 is minimal fluorescence yield when the PSII reaction center is open. The Fm is maximal fluorescence yield at the time of closure of PSII reaction center. The Fm´ is the maximal fluorescence in the light-adapted state. The Fv/Fm is the photochemical efficiency of PS II and is used as basic tool in plant photosynthetic activity. In order to measure these parameters, expanded leaves were first dark adaptation for 15 min. Then, all measurements were taken from the same leaf. There were three replications and then three leaves were randomly selected of three different plants from each replication. Measurements for each parameter on a single leaf were done at five different locations and their means were calculated. Thus, for every replication, the means were calculated for 15 different locations of the three different leaves.

### Determination of relative electrolyte leakage (REL)

Plasma membrane integrity in roots was assessed in terms of relative electrolyte leakage (REL). Root tissues (100 mg) were cut into small pieces and vibrated for 30 min in deionized water followed by measurement of conductivity of bathing medium (EC_1_). The samples were again boiled for 15 min and second conductivity was measured (EC_2_) [27]. Total electrical conductivity was calculated by using the following formula.

$$\text{REL }\left(\text{\%}\right)\;=\;\left(\frac{\text{EC}1}{\text{EC}2}\right)\times \;100$$

### Determination of antioxidant enzyme activities

For enzyme activities, leaf samples (0.5 g) were homogenized in 8 mL of 50 mM potassium phosphate buffer (pH 7.8) under ice cold conditions. Homogenate was centrifuged at 10,000g for 20 min at 4°C and the supernatant was used for the determination of the following enzyme activities. The assay for ascorbate peroxidase (APX, EC 1.11.1.11) activity was measured in a reaction mixture of 3 mL containing 100 mM phosphate (pH 7), 0.1 mM EDTA-Na_2_, 0.3 mM ascorbic acid, 0.06 mM H_2_O_2_ and 100 μL protein extract. After 30 s of addition of H_2_O_2_, change in absorption was taken at 290 nm [28]. Catalase (CAT, EC 1.11.1.6) activity was measured according to Aebi [29] with the use of H_2_O_2_ (extinction co-efficient 39.4 mM cm^-1^) for 1 min at A240 in 3 mL reaction mixture containing 50 mM potassium phosphate buffer (pH 7.0), 2 mM EDTA-Na_2_, 10mM H_2_O_2_ and 100 μL protein extract. Glutathione reductase (GR, EC 1.6.4.2) activity was assayed by Jiang and Zhang [30], where NADPH oxidation was followed at 340 nm. The reaction mixture was comprised of 50 mM potassium phosphate buffer (pH 7.0), 2 mM EDTA-Na_2_, 0.15 mM NADPH, 0.5 mM GSSG and 100 μL enzyme extract in a 1 mL volume. The reaction was started by using NADPH.

Total superoxide dismutase (SOD, EC 1.15.1.1) activity was determined with the method of Zhang et al. [31] following the inhibition of photochemical reduction due to nitro blue tetrazolium (NBT). The reaction mixture was comprised of 50 mM potassium phosphate buffer (pH 7.8), 13 mM methionine, 75 μM NBT, 2 μM riboflavin, 0.1 mM EDTA and 100 μL of enzyme extract in a 3-mL volume. One unit of SOD activity was measured as the amount of enzyme required to cause 50% inhibition of the NBT reduction measured at 560 nm. Peroxidase (POD, EC1.11.1.7) activity was assayed by Zhou and Leul [32] with some modifications. The reactant mixture contained 50 mM potassium phosphate buffer (pH 7.0), 1% guaiacol, 0.4% H_2_O_2_ and 100 μL enzyme extract. Variations due to guaiacol were measured at 470 nm.

### Total RNA extraction and gene expression analysis

Total RNA was extracted from ~100 mg of leaf and root tissues using manual (Trizol) method. Prime Script RT reagent kit (Takara Co. Ltd) with gDNA eraser was used to remove the genomic DNA and cDNA synthesis. cDNA samples from different treatments were assayed by quantitative real time PCR (RT-qPCR) in the iCycler iQ Real-time detection system (Bio-Rad, Hercules, CA, USA) using SYBRPremix Ex Taq II (Takara Co. Ltd). The PCR conditions consisted denaturation at 95°C for 3 min, followed by 40 cycles of denaturation at 95°C 30 s, annealing at 58°C for 45 s and finally extension at 72 s for 45 s. Gene-targeting primers were designed based on mRNA or expressed sequence tag (EST) for the corresponding genes as follows: SOD (F: 5′ ACGGTGTGACCACTGTGACT 3′, R: 5′ GCACCGTGTTGTTTACCATC3′), POD (F: 5′ATGTTTCGTGCGTCTCTGTC3′, R: 5′ TACGAGGGTCCGATCTTAGC 3′), CAT (F: 5′ TCGCCATGCTGAGAAGTATC 3′, R: 5′ TCTCCAGGCTCCTTGAAGTT 3′), APX (F:5′ ATGAGGTTTGACGGTGAGC 3′, R:5′ CAGCATGGGAGATGGTAGG 3′), GR (F: 5′ AAGCTGGAGCTGTGAAGGTT 3′, R: 5′ AGACAGTGTTCGCAAAGCAG 3′), and Actin gene (F: 5′ TTGGGATGGACCAGAAGG 3′, R: 5′ TCAGGAGCAATACGGAGC 3′) as an internal control. The software given with the PCR system was used to calculate the threshold cycle values and quantification of mRNA levels was performed according to the method of Livak and Schmittgen [33]. The threshold cycle (Ct) value of actin was subtracted from that of the gene of interest to obtain ΔCt value. *Brassica napus* actin gene 2.1 (FJ529167) was used as an internal control.

### Protein extraction, visualization and identification studies

#### Soluble protein extraction and 2-DE analysis

For proteomic study, two biological independent replicates were used throughout the experiment. Total soluble proteins were extracted as described by Carpentier et al. [34] with minor modification using phenol extraction method. Concentration was determined as according to Bradford [35]. Proteins were separated by two-dimensional gel electrophoresis (2-DE), and the protein spots in analytical gels were visualized by silver staining method.

#### Protein visualization, image analysis, and quantification

Protein visualization, image analysis, and quantification were done as followed by Bah et al. [36]. Briefly, PowerLook1100 scanner (UMAX) was used for scanning and calibration of the protein spots. Only those with significant and reproducible changes (*P*≤0.05) were considered to be differentially accumulated proteins. The target protein spots were automatically excised from the stained gels and digested with trypsin using a Spot Handling Workstation (Amersham Biosciences). Peptides gel pieces were placed into the EP tube and washed with 1:1 mixture of 50 μL of 30 mM K_3_Fe(CN)_6_ and 100 mM NaS_2_O_3_ for 10–15 min until completely discolored then washed with 200 μL bi-distilled water (two times for 5 min each). The washed solution was drained and gel slices washed with 50% CAN (acetonitrile, Fisher A/0626/17) and 100% ACN rotationally, and then incubated in 25 mM NH_4_HCO_3_ (Sigma A6141) for 5 min at 37°C. After absorbance of incubation solvent, 50% ACN and 100% ACN was rotationally added and dried at 40°C for 5 min respectively.

Trypsin digestion was carried out as follows: sequencing-grade porcine trypsin (Promega, Madison, WI, USA) was suspended in 25 mM NH_4_HCO_3_ at a concentration of 12.5ng/μL used to rehydrate the dried gel pieces. The trypsin digestion was carried out for 16 hr at 37°C. Peptides were extracted from the digest as follows for three times: 10 μL of 50% ACN containing 0.1% TFA (trifluoroacetic acid, GE HealthCare) was added to each tube and incubated for 5 min at 37°C and transferred the supernatants to new EP tube. The extracts were pooled and then vacuum concentrated for about 2 hr. A solution of peptides was filtrated via Millipore (Millipore ZTC18M096) and mixed with the same volume of a matrix solution consisting of saturated α-cyano-4-hydroxycinnamic acid (CHCA) in 50% ACN containing 0.1% TFA.

After the peptides were co-crystallized with CHCA by evaporating organic solvents, tryptic-digested peptide masses were measured using a MALDI-TOF-TOF mass spectrometer (ABI4700 System, USA). All mass spectra were recorded in positive reflector mode and generated by accumulating data from 1000 laser shots. The following threshold criteria and settings were used: detected mass range of 700–3200 Da (optimal resolution for the quality of 1500 Da), using a standard peptide mixture (des-Argl-Bradykinin Mr904.468, Angiotensin I Mr1296.685, Glul-Fihrinopeptide B Mr1570.677, ACTH (1–17) Mr2093.087, ACTH (18–39) Mr2465.199; ACTH (7–38) Mr3657.929) as an external standard calibration, with laser frequency of 50 Hz, repetition rate of 200 Hz, UV wavelength of 355 nm, and accelerated voltage of 20,000 V. Peptide mass fingerprint data were matched to the NCBInr database using Profound program under 50 ppm mass tolerance.

#### Peptide and protein identification by database search

Data were processed via the Data Explorer software and proteins were unambiguously identified by searching against a comprehensive non-redundant sequence database using the MASCOT software search engine (http://www.matrixscience.com/cgi/search%20form.pl?FORMVER=2&SEARCH=MIS). Moreover, in order to evaluate protein identification, we considered the percentage of sequence coverage, the observation of distribution of matching peptides (authentic hit is often characterized by peptides that are adjacent to one another in the sequence and that overlap), the distribution of error (distributed around zero), the gap in probability and score distribution from the first to other candidate; only matches with over 90% sequence identity and a maximum e-value of 10^-10^ were considered.

### Statistical analysis

The data was analyzed by using a statistical package, SPSS version 16.0 (SPSS, Chicago, IL, USA). A two-way variance analysis (ANOVA) was carried out, followed by the Duncan’s multiple range test.

## Results

### ALA improves metabolic changes under Cd stress

**Fig 1:**
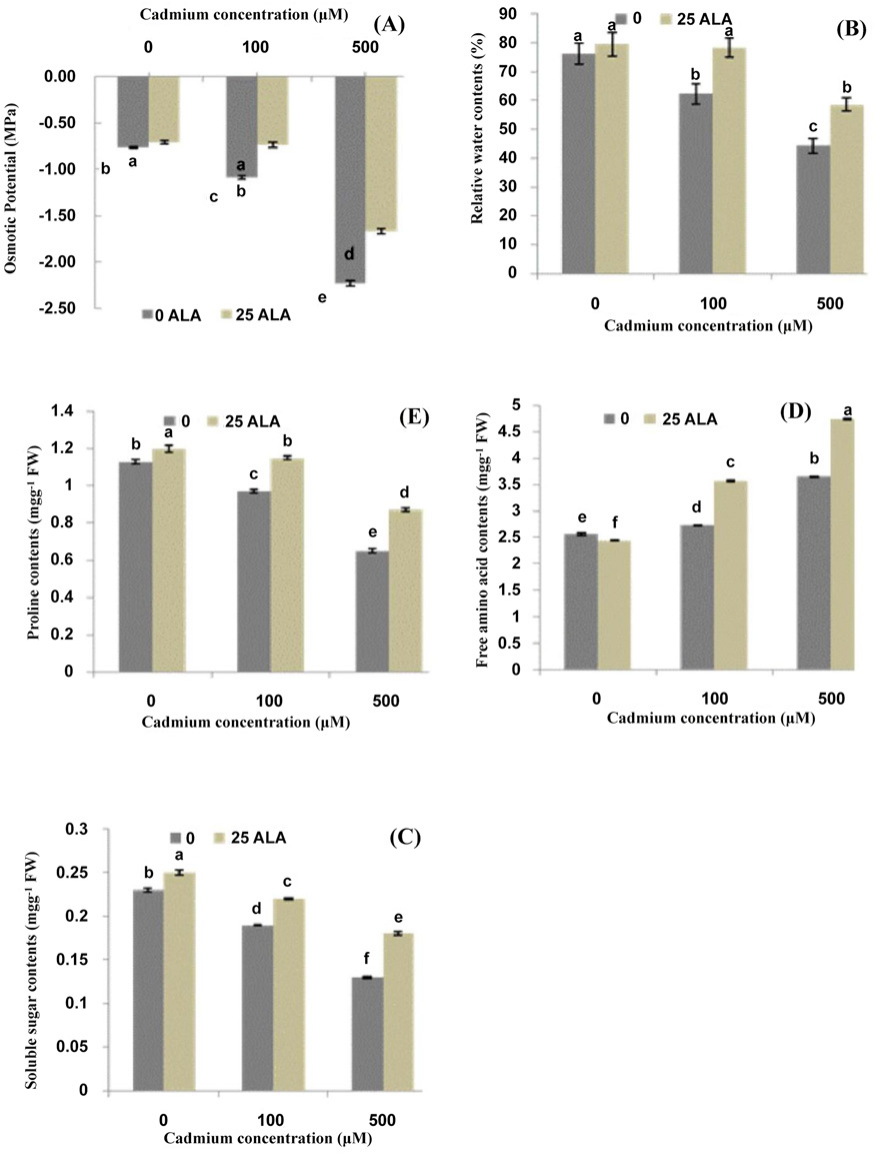
Effects of different treatments of 5-aminolevulinic acid (ALA) (mg/L) and cadmium (Cd^2+^) (μM) on (A) osmotic potential (MPa), (B) relative water contents (%), (C) soluble sugar contents (mg g^-1^ FW), (D) free amino acid contents (mg g^-1^ FW) and (E) proline contents (mg g^-1^ FW) in the leaves of *Brassica napus* cv. ZS758. Values are the means ± SD of three replications. Variants possessing the same letter are not statistically significant at P < 0.05.

The present study demonstrated that leaf osmotic potential and relative water contents decreased with an increase in cadmium (Cd^2+^) concentrations (Fig 1A and 1B). However, exogenous application of ALA helped the plants to adjust osmotic potential and relative water contents. The plants treated with 100 μM Cd^2+^ recovered completely, and those treated with 500 μM also significantly recovered with the application of ALA. Results showed that Cd^2+^ stress alone significantly lowered the soluble sugar contents in *B*. *napus* leaves as compared to control (Fig 1C). This decrease was (18% and 43%) under different treatments of Cd^2+^ (100 and 500 μM) respectively as compared to control plants. Meanwhile, foliar application of ALA significantly improved soluble sugar contents in *B*. *napus* leaves under both Cd^2+^ stress levels. Moreover, the plants treated with Cd^2+^ stress alone showed a significant increase in free amino acids contents in *B*. *napus* leaves (Fig 1D). At the same time, exogenously applied ALA showed a synergetic affect and it further improved the free amino acids contents in the leaves of *B*. *napus* under Cd^2+^ stress. Moreover, it was found that that Cd^2+^ stress alone significantly reduced the proline contents in *B*. *napus* leaves (Fig 1E). Besides, the application of ALA significantly improved the proline contents in the leaves of *B*. *napus* under different Cd^2+^ stress levels. Moreover, ALA alone significantly enhanced the proline contents in the leaves of *B*. *napus* as compared to control plants.

### ALA up-regulates Cd-induced fluorescence parameters and down-regulates REL

**Fig 2:**
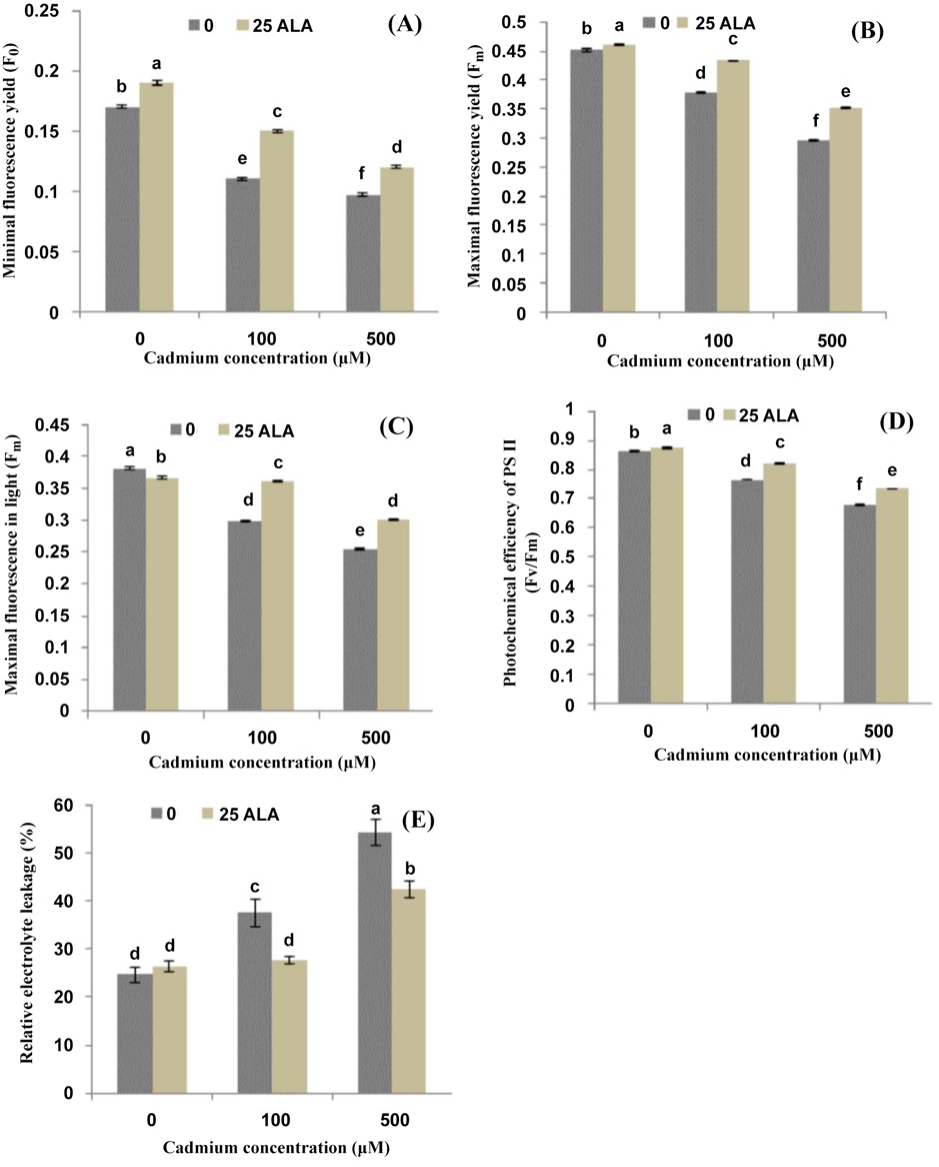
Effects of different treatments of 5-aminolevulinic acid (ALA) (mg/L) and cadmium (Cd^2+^) (μM) on photochemical quenching parameters (A) minimal fluorescence yield (F_0_), (B) maximal fluorescence yield (F_m_), (C) maximal fluorescence in light (F_m´_), (D) photochemical efficiency of PS II (Fv/Fm) and (E) relative electrolyte leakage (%) in the leaves of *Brassica napus* cv. ZS758. Values are the means ± SD of three replications. Variants possessing the same letter are not statistically significant at P < 0.05.

Effects of different treatments of ALA and Cd^2+^ on chlorophyll fluorescence of photosynthetic machinery are presented in Fig 2. Results revealed that Cd^2+^ stress alone significantly reduced the photosynthetic quenching parameters such as F0, Fm, Fm´ and Fv/Fm as compared to control. The higher concentration of Cd^2+^ (500 μM) decreased F0 by (43%), Fm by (34%), Fm´ by (33%) and Fv/Fm by (22%) as compared to control. However, foliar application of ALA reduced the deleterious effects of Cd and significantly improved these parameters as compared to their respective controls. Furthermore, application of ALA alone also increased these parameters except Fm´ as compared to control plants. The present study also showed that REL increased linearly in the leaves of *B*. *napus* as we increased Cd^2+^ stress alone in the solution (Fig 2E). The increase in REL was (53% and 120%) under different treatments of Cd^2+^ (100 and 500 μM) respectively as compared to control plants. Foliar applied ALA significantly reduced the REL in *B*. *napus* under Cd^2+^ stress. However, ALA alone did not show any significant change on the contents of REL of *B*. *napus* as compared to control plants (Fig 2E).

### ALA alleviates Cd-induced antioxidants activities and their transcript levels

**Fig 3:**
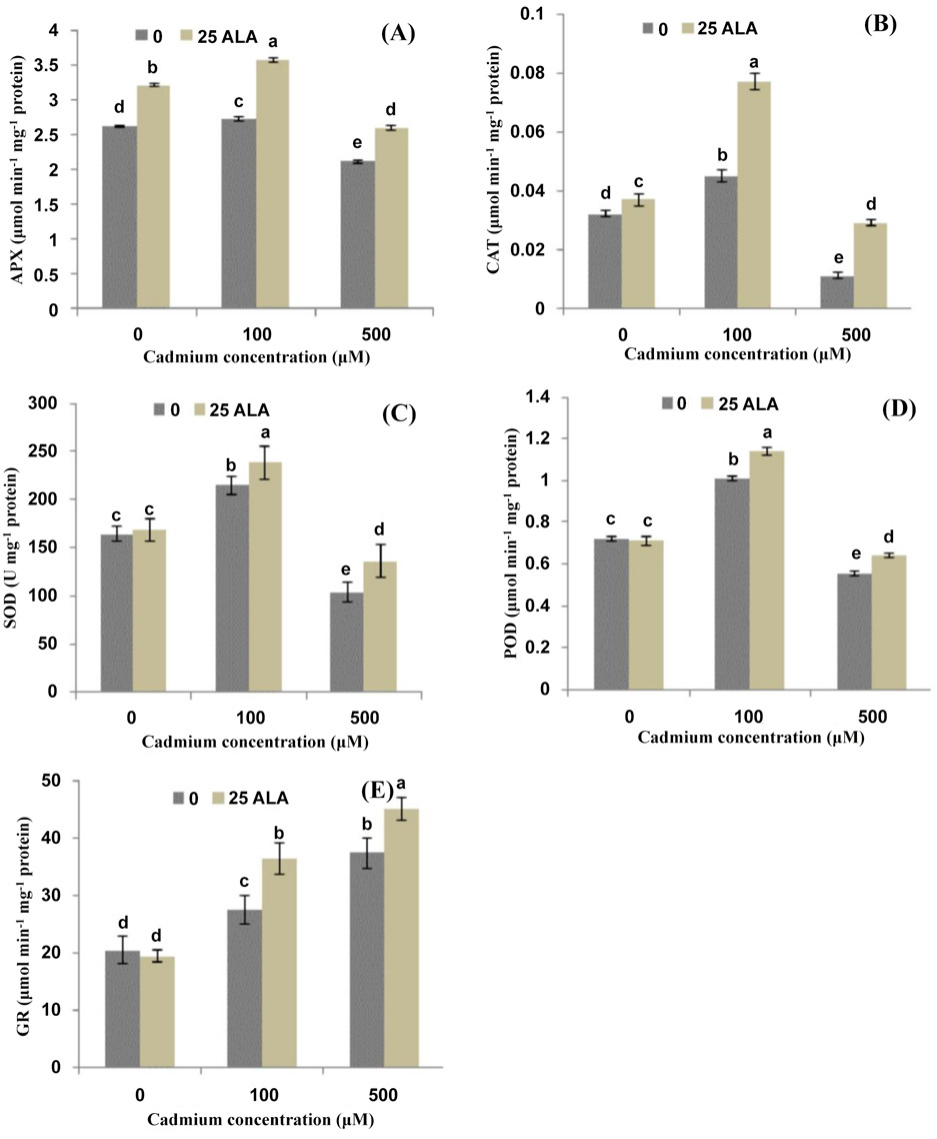
Effects of different treatments of 5-aminolevulinic acid (ALA) (mg/L) and cadmium (Cd^2+^) (μM) on the activities of (A) ascorbate peroxidase (APX), (B) catalase (CAT), (C) superoxide dismutase (SOD), (D) peroxidase (POD) and (E) glutathione reductase (GR) in the leaves of *Brassica napus* cv. ZS 758. Values are the means ± SD of three replications. Variants possessing the same letter are not statistically significant at P < 0.05.

**Fig 4:**
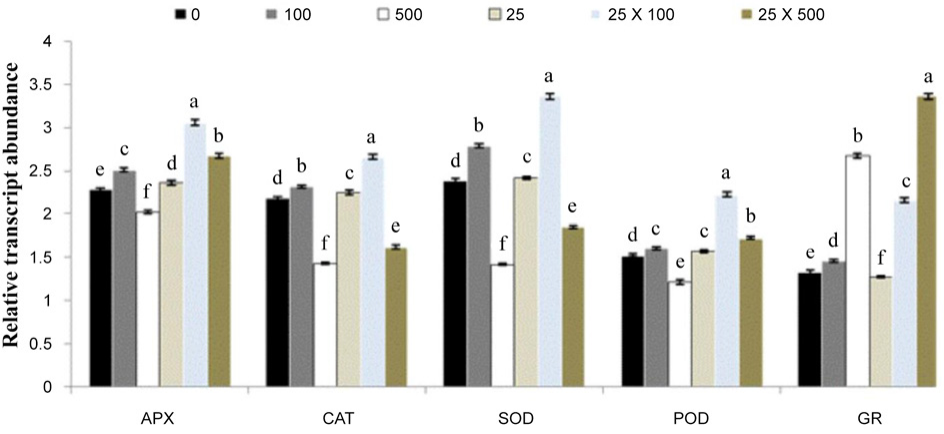
Effects of different treatments of 5-aminolevulinic acid (ALA) (mg/l) and cadmium (Cd^2+^) (μM) on the expression of ascorbate peroxidase gene (APX), catalase gene (CAT), superoxide dismutase gene (SOD), peroxidase gene (POD) and glutathione reductase gene (GR) in the leaves of *Brassica napus* cv. ZS758. The RT-qPCR analysis was performed to examine mRNA levels of five antioxidant enzyme genes in plants treated with CK, 100 μM Cd^**2+**^ alone, 500 μM Cd^**2+**^ alone, 25 mg/l ALA alone, 100 μM Cd^**2+**^ + 25 mg/l ALA and 500 μM Cd^**2+**^ + 25 mg/l ALA. Values are the means ± SD of three replications. Means followed by the same letter did not significantly differ at P<0.05 according to Duncan’s multiple range test.

Effects of different treatments of ALA and Cd^2+^ on antioxidant enzyme activities in the leaves of *B*. *napus* are shown in Fig 3. Results showed that lower level of Cd^2+^ alone (100 μM) significantly increased the activities of APX and CAT in *B*. *napus* leaves as compared to control (Fig 3A and 3B). However, a significant decrease was found under the higher concentration of Cd^2+^ (500 μM). The application of ALA alleviates the Cd stress and improved APX and CAT activities significantly in *B*. *napus* leaves. Exogenous ALA increased the activity of APX by (30% and 36%) and CAT by (71% and 163%) under 100 and 500 μMCd^2+^ respectively, as compared to their respective controls. Results showed that lower level of Cd^2+^ alone showed a stimulatory effect on the activities of SOD and POD, while, higher level of Cd^2+^ alone showed inhibitory effect on SOD and POD activities (Fig 3C and 3D). Under Cd^2+^ stress conditions, ALA induced a significant increase in the activity of SOD and POD and this increase was 18% and 30% for SOD and 13% and 17% for POD under 100 and 500 μMCd^2+^stress respectively, as compared to their respective controls. The data depicted that activity of GR showed an increasing trend with an increase in different Cd^2+^ concentrations (Fig 3E). Exogenously applied ALA showed a synergetic effect on GR activity and increased the contents under different Cd^2+^ levels. Moreover, the present study also depicted that application of ALA alone did not show any significant change in the activities of SOD, POD and GR; however, ALA enhanced the APX and GR activities significantly as compared to control (Fig 3). To analyze the effect of ALA on gene expression in *B*. *napus* leaves under Cd stress, we examined five antioxidant enzyme genes APX, CAT, SOD, POD and GR. As shown in Fig 4, lower level of Cd^2+^ significantly increased the expression of APX, CAT, SOD and POD; however, higher level significantly induced these gene expressions as compared to control. The expression level of all the antioxidant enzymes was also up-regulated with the application of ALA under both Cd stress conditions (Fig 4).

### Cd-induced proteomic changes in *B*. *napus* leaves and alleviation through ALA

#### Classification of identified proteins spots

**Fig 5:**
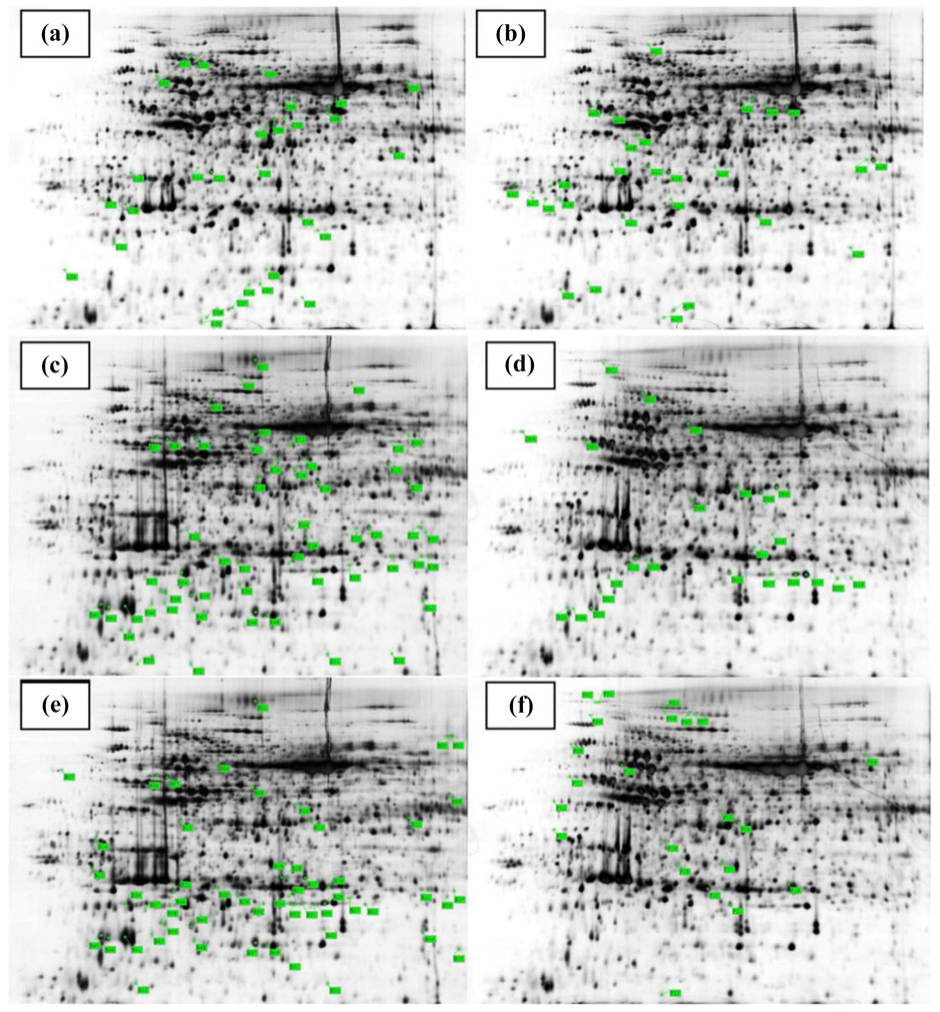
Representative 2-DE maps comparing *Brassica napus* cv. ZS 758 leaf proteins isolated from normal (a, d) and under 500 μM Cd^2+^ alone (b, e) and under combine application of ALA and 500 μM Cd^2+^ (c, f) along with protein marker. Differentially accumulated protein spots are indicated by green sashes. Twenty-nine down-regulated spots (C01-C29) and twenty-four up-regulated spots (D01-D24) under control conditions (a, d); twenty-seven down-regulated spots (A01-A27) and fifty-three up-regulated spots (B01-B53) under Cd^**2+**^ alone (b, e); fifty-three down-regulated spots (E01-E53) and twenty-two up-regulated spots (F01-F22) under the combine treatment of ALA and Cd^**2+**^ (c, f) are indicated on the map.

In order to investigate, how ALA alleviates the Cd^2+^-induced proteomic changes, total crude proteins were extracted for 2-D gel electrophoresis (Fig 5). Based on 1.5-fold quantitative change criteria for expression, total thirty-four protein spots showed differential expression between Cd and/or ALA-treated leaves proteomes of *B*. *napus* (S1 and S2 Figs). Eighteen spots contained down-regulated proteins (Table 1) and sixteen spots showed up-regulated proteins (Table 2). Out of thirty four differentially regulated proteins, seventeen (50%) showed no matches, while, other seventeen (50%) could be identified by proteomics analysis (S1 Fig). Out of seventeen identified reproducible protein spots, seven proteins (41%) were induced and ten proteins (59%) were up-regulated. Out of seventeen unmatched proteins, eleven proteins (64%) proteins were from down-regulated category and the rest six proteins (36%) was from up-regulated category. Moreover, data showed that out of 18 down regulated proteins, six proteins were found at Cd^2+^ alone conditions (S1 Fig A lettering) and seven proteins were found at the combine treatment of ALA and Cd^2+^ as compared to Cd^2+^ alone conditions (S1 Fig E lettering) as well as five proteins were obtained at the combine treatment of ALA and Cd^2+^ as compared to control (S1C Fig lettering). However, out sixteen up-regulated proteins, ten proteins were found at Cd^2+^ alone conditions as compared to control (S2B Fig lettering) and five proteins were found at the combine treatment of ALA and Cd^2+^ as compared to Cd^2+^ alone conditions (S2F Fig lettering) as well as only one protein was observed at the combine treatment of ALA and Cd^2+^ as compared to control (S2D Fig lettering).

**Table 1:** Down-regulated proteins in the leaves of *Brassica napus* cv. ZS 758 under 500 μM Cd^2+^ alone as compared to control (with A lettering), under the combine treatment of ALA and 500 μM Cd^2+^ as compared to Cd^2+^ alone (with E lettering) and under the combine treatment of ALA and 500 μM Cd^2+^ (with C lettering) as compared to control conditions.

Spot ID	Acc. No	Homologous and specie name	Mascot score	Mol. Mass	IP Value	Peptides match	Fold decrease	Functions
A04	gi|557102603	hypothetical protein EUTSA_v10013710mg (*Eutrema salsugineum*)	50	44539	5.74	1	1.8	
A05	gi|557092637	hypothetical protein EUTSA_v10004217mg (*Eutrema salsugineum*)	141	48063	6.52	10	2.1	
A06	gi|330254622	ribulose bisphosphate carboxylase/oxygenase activase (*Arabidopsis thaliana*)	158	48754	7.55	14	2.5	CO_2_ assimilation/photosynthesis
A13	gi|557099707	hypothetical protein EUTSA_v10014179mg (*Eutrema salsugineum*)	213	33483	5.79	16	1.9	
A18	gi|12230623	Ribonucleoprotein At2g37220, chloroplastic; Precursor	79	30699	5.06	5	2.0	Protein synthesis/regulation
A24	gi|75161441	30S ribosomal protein S6 alpha	65	22975	5.92	4	10	Protein synthesis/regulation
E05	gi|482570957	hypothetical protein CARUB_v10020280mg (*Capsella rubella*)	113	50895	5.85	7	10	
E20	gi|75205048	Thioredoxin-like protein, Chloroplastic drought-induced stress protein	121	33948	8.65	9	1.9	Stress related
E24	gi|557099778	hypothetical protein EUTSA_v10014474mg (*Eutrema salsugineum*)	108	27210	6.77	6	10	
E25	gi|557099778	hypothetical protein EUTSA_v10014474mg (*Eutrema salsugineum*)	177	27210	6.77	5	10	
E29	gi|557115451	hypothetical protein EUTSA_v10025815mg (*Eutrema salsugineum*)	30	33651	9.24	1	1.8	
E48	gi|557095024	hypothetical protein EUTSA_v10008422mg (*Eutrema salsugineum*)	216	30292	8.5	15	10	
E52	gi|482572100	hypothetical protein CARUB_v10010538mg (*Capsella rubella*)	152	17198	5.24	6	10	
C02	gi|482565882	hypothetical protein CARUB_v10013177mg (*Capsella rubella*)	180	71578	5.04	17	10	
C03	gi|30316342	2,3-bisphosphoglycerate-independent phosphoglycerate mutase 1	230	60770	5.32	10	10	Carbohydrate metabolism
C14	gi|17380545	Proteasome subunit alpha type-1-A	114	30685	4.99	6	10	Dehydration of damaged protein
C15	gi|2501188	Thiamine thiazole synthase	266	36755	5.82	9	1.9	Catalysis
C26	gi|557107168	hypothetical protein EUTSA_v10021585mg (*Eutrema salsugineum*)	75	40658	4.45	8	10	

**Table 2:** Up-regulated proteins in the leaves of *Brassica napus* cv. ZS 758 under 500 μM Cd^2+^ alone as compared to control (with B lettering), under the combine treatment of ALA and 500 μM Cd^2+^ as compared to Cd^2+^ alone (with F lettering) and under the combine treatment of ALA and 500 μM Cd^2+^ (with D lettering) as compared to control conditions.

Spot ID	Acc. No	Homologous and specie name	Mascot score	Mol. Mass	IP Value	Peptides match	Fold increase	Function
B11	gi|211905345	epithiospecifier protein (*Brassica rapa* subsp. pekinensis)	245	37890	5.95	19	10	Carbohydrate metabolism
B20	gi|557094682	hypothetical protein EUTSA_v10008467mg (*Eutrema salsugineum*)	74	30337	7.6	7	10	
B21	gi|266891	Ribulose bisphosphate carboxylase small chain	330	20499	8.23	8	1.7	CO_2_ assimilation/photosynthesis
B24	gi|557114672	hypothetical protein EUTSA_v10027518mg (*Eutrema salsugineum*)	30	27724	6.86	1	2.0	
B27	gi|557102656	hypothetical protein EUTSA_v10014706mg (*Eutrema salsugineum*)	161	21846	6.1	4	10	
B28	gi|544370614	iron superoxide dismutase (*Brassica rapa* subsp. oleifera)	67	23833	5.96	5	2.9	Defense
B39	gi|227438283	disease resistance protein (*Brassica rapa* subsp. pekinensis)	19	142209	6.48	1	4.7	Stress related
B45	gi|17369187	Regulator of ribonuclease-like protein 1	164	18110	5.68	5	5.1	Protein synthesis/ Regulation
B47	gi|332646777	peptidyl-prolyl cis-trans isomerase CYP20-3 (*Arabidopsis thaliana*)	165	34696	9.24	7	5.3	Redox homeostasis
B53	gi|22001642	Non-symbiotic hemoglobin 2	81	18364	5.89	7	2.3	Transport protein
F09	gi|544604537	Dihydrolipoyl dehydrogenase 1	79	66905	8.56	10	1.8	Carbohydrate metabolism
F11	gi|557104099	hypothetical protein EUTSA_v10005987mg (*Eutrema salsugineum*)	206	48751	5.08	5	1.8	
F14	gi|557089213	hypothetical protein EUTSA_v10011629mg (*Eutrema salsugineum*)	261	38323	5.88	16	1.7	
F17	gi|15218330	light harvesting complex photosystem II subunit 6 (*Arabidopsis thaliana*)	37	27505	6.75	1	1.8	CO_2_ assimilation/photosynthesis
F19	gi|75161441	30S ribosomal protein S6 alpha	69	22975	5.92	4	10	Protein synthesis/ Regulation
D04	gi|557112823	hypothetical protein EUTSA_v10025445mg (*Eutrema salsugineum*)	75	40658	4.45	8	10	

#### Functional distribution of identified proteins

**Fig 6:**
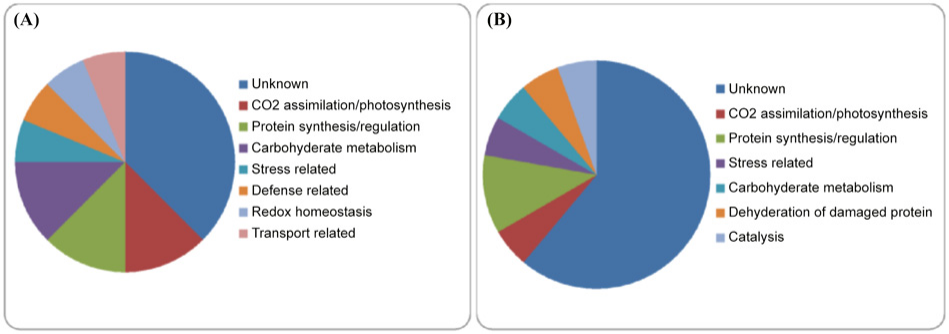
Differentially regulated *Brassica napus* proteins due to Cd^2+^ and/or ALA treatments. **(A)** Functional distribution of the 16 up-regulated proteins that were differentially produced in response to Cd^**2+**^ and/or ALA treatments.**(B)** Functional distribution of the 18 down-regulated proteins that were differentially produced in response to Cd^**2+**^ and/or ALA treatments.

Out of thirty four detected proteins, seventeen Cd^2+^ and/or ALA-responsive proteins were identified in the treated leaves. They are involved in different biological functions and pathways during the cellular adaptation to Cd^2+^ and/or ALA. All the identified proteins were laid under different functional classes on the basis of their putative functions (Fig 6A and 6B). These pathways and biological functions are such as CO_2_ assimilation/ photosynthesis, protein synthesis/ regulation, stress related, carbohydrate metabolism, dehydration of damaged proteins, catalysis, defense, redox homeostasis and transport proteins. The identified proteins belong to different functional groups. The functional groups are CO_2_ assimilation/ photosynthesis (18%, designated as A06, B21, F17), protein synthesis/ regulation (23% designated as A18, A24, B45, F19), stress related (11%, designated as E21, B39), carbohydrate metabolism (18%, designated as C03, B11, F07), dehydration of damaged proteins (6%, designated as C14), catalysis (6%, designated as C15), defense related proteins (6%, designated as B28), redox homeostasis (6%, designated as B47) and transport proteins (6%, designated as B53) (Table 1 and 2).

#### Identified proteins under Cd stress conditions

Regarding the Cd^2+^ stress alone conditions, the identified induced proteins belonged two main functional categories i.e. CO_2_ assimilation/ photosynthesis and protein synthesis/ regulation (Table 1). The proteins of these two categories (A06, A18, A24) were significantly down-regulated under Cd^2+^ alone conditions as compared to control (S1 Fig). The CO_2_ assimilation/ photosynthesis related protein was “ribulose bisphosphate carboxylase” (gi|330254622), which uses in the photosynthesis process in plants. However, protein synthesis/ regulation related protein were “ribonucleo protein” (gi|12230623) and “ribosomal protein” (gi|75161441) which is involved in protein synthesis.

The identified up-regulated proteins under Cd^2+^ alone conditions came under five main functional categories i.e. carbohydrate metabolism, CO_2_ assimilation/ photosynthesis, defense related, stress related and protein synthesis/ regulation (Table 2). The proteins of these five categories (B11, B21, B28, B39, B45) were significantly up-regulated under Cd^2+^ alone conditions as compared to control (S2 Fig). The carbohydrate metabolism and CO_2_ assimilation/ photosynthesis related protein were “epithiospecifier protein” (gi|211905345) and “ribulose bisphosphate carboxylase” (gi|266891) respectively, which are involved in the metabolism and assimilation of proteins. Other proteins such as “iron superoxide dismutase” (gi|544370614), “disease resistance protein” (gi|227438283) and “regulator of ribonuclease” (gi|17369187) were activated during the defense, stress and regulation of proteins, respectively.

#### ALA alleviates proteomic changes under Cd stress

Under the combine treatment of ALA and Cd^2+^ tress, among the identified down-regulated proteins, identified proteins were found under four main functional categories i.e. stress related, carbohydrate metabolism, dehydration of damaged proteins and catalysis (Table 1). There was only one protein of stress related category (E20), which was significantly down-regulated under the combine treatment of ALA and Cd^2+^ as compared to Cd^2+^ alone stress conditions. This protein is “thioredoxin-like protein” (gi|75205048), which uses in the plants against the stress conditions. However, carbohydrate metabolism, dehydration of damaged proteins and catalysis related protein were “2,3-bisphosphoglycerate-independent phosphoglycerate mutase 1” (gi|30316342), proteasome (gi|17380545) and “thiamine thiazole synthase” (gi|2501188) respectively. These three proteins (C03, C14, C15) were significantly down-regulated under the combine treatment of ALA and Cd as compared to control conditions (S1 Fig).

Among the identified up-regulated proteins under the combine treatment of ALA and Cd^2+^, identified proteins were from two main functional catagories i.e. CO_2_ assimilation/ photosynthesis and protein synthesis/ regulation (Table 2). The proteins of these two categories (F17, F19) were significantly up-regulated during the combine treatment of ALA and Cd as compared to Cd alone conditions (S2 Fig). These proteins were “light harvesting complex photosystem II subunit 6” (gi|15218330) and “30S ribosomal protein S6 alpha” (gi|75161441) respectively, which used in the photosynthesis and regulations of proteins in the leaves.

## Discussion

### ALA alleviates Cd stress and improves metabolic changes in *B*. *napus*

Cadmium (Cd) stress can also be attributed to the water stress or a kind of physiological drought generated by Cd^2+^ [37], as evident from the decrease in osmotic potential and relative water content (Fig 1). The decline in osmotic potential in the Cd^2+^ solution resulted from reduced tissue water content, and some entry of sodium (Na^+^) and chloride (Cl^-^) solutes with maintenance of K^+^ concentrations and increased production of organic solutes. ALA application induced an increase in osmotic potential and relative water contents of the stressed seedlings. Our findings are similar with those of Naeem et al. [38], who found that ALA increased osmotic potential and relative water contents under salinity stress in *B*. *napus* leaves. The accumulation of compatible solutes at high concentrations is believed to facilitate ‘‘osmotic adjustment”, can reduce inhibitory effects of ions on enzyme activity and prevent dissociation of enzyme complexes [39]. Proline, soluble sugars and free amino acids have been mentioned as important compatible solutes in osmoregulation and protect plants from stress through different mechanisms, including cellular osmotic adjustment, detoxification of reactive oxygen species, protection of membrane integrity and stabilization of proteins/ enzymes [40]. Some researchers correlate the accumulation of proline under stress as stress tolerance [41], while others consider the accumulation of proline as stress injury [42]. The present study authenticates the findings of Nayyar and Walia [41], as results show that foliar application of ALA enhanced proline accumulation in the leaves under salinity stress (Fig 1), as reported in *Brassica juncea* seedlings [43].

Free amino acid (FAA) accumulation in plants under Cd^2+^ stress has often been attributed to alterations in biosynthesis and degradation processes of amino acids and proteins [44]. In this study, FAA content increased in leaves (Fig 1) under Cd^2+^ stress alone or in combination with ALA. So, it can hypothesize that ALA treatment may stimulate hydrolysis of proteins, providing a pool of compatible osmolyte, which is important in osmotic adjustment [18]. This hypothesis could be supported by the observation that ALA increased FAA accumulation at the sake of proteins.

### ALA alleviates Cd-induced photosynthetic fluorescence parameters

This study depicted that Cd^2+^ stress significantly decreased the photosynthetic fluorescence pigments (Fig 2). Previously, decrease in chlorophyll pigments under Cd^2+^ stress was also found in *Brassica* [6]. Such inhibition of biosynthesis might be the key factor for reduced photosynthesis and growth by Cd^2+^ [16]. Up-regulation of ROS and MDA under heavy metal stress might have caused the disturbances in the electron transport rates of PSI and PSII [45]. Another reason for such decline might be the indirect interaction of Cd^2+^ with photosynthesis by competing for root absorption with other metals (Mg, Fe, Mn, Zn) that are essential cofactors of enzymes, pigments and structure components of the photosynthetic apparatus [4].

It has been reported that exogenous application of ALA (a key precursor of chlorophyll) can induce the development of chloroplasts, which is reflected by the raised fluorescence pigments in the present study (Fig 2). These results are consistent with those of Wang et al. [46]; who reported that ALA may regulate the biosynthesis of chlorophyll. The effectiveness of ALA in enhancing photosynthetic efficiency can therefore be attributed to the significant improvement of fluorescence pigments and subsequently boosting light-harvesting capabilities of the treated plants [38]. Moreover, present findings suggested that Cd^2+^ stress displayed a negative effect on membrane integrity and thus deterioration of membranes occurred and that’s why relative electrolyte leakage enhanced under the Cd^2+^ toxicity (Fig 2E). The possible reason is that under heavy metal stress, plants have evolved the antioxidant enzymes mechanism that act to detoxify the changes caused by reactive oxygen species and maybe due to that this increase was occurred [47]. However, foliar applied ALA regulates the Cd^2+^ stress and decreased the REL in *B*. *napus* leaves. These findings are in line with those of Yusuf et al. [43] in *Brassica juncea* and El-Tayeb [48] in barley under NaCl stress. However, foliar application of ALA decreased the REL by improving Cd^2+^ tolerance; as reported by Naeem et al. [11] in *B*. *napus*.

### ALA regulates Cd-induced antioxidant machinery

According to present study, APX, CAT, SOD and POD activities decreased under higher concentration of Cd^2+^ (500 μM); however, GR activity was increased under different Cd^2+^ concentrations (Fig 3). Similar to these results, such reduction in SOD activity under Cd^2+^ stress was reported previously in wheat [49] and in pea [50]. In the present study, the antioxidant enzymes i.e. APX, CAT, POD, and SOD enhanced their activities under the combined application of ALA and Cd^2+^ at different concentrations (Fig 3). This may be due to the fact that ALA is an essential precursor of heme-based molecules; its application can increase the activity of these bio-molecules (APX, POD and CAT) and helps scavenge the H_2_O_2_ to provide protection against harmful effects caused by ROS under Cd^2+^ stress [6]. Moreover, Liu et al. [51] and Naeem et al. [18] also observed an enhancement in antioxidant enzyme activities with the application of ALA under the water-deficit and salinity stress respectively in the leaves of *B*. *napus*.

In the present study, the expression of antioxidant genes (APX, CAT, SOD, POD and GR) was also examined (Fig 4). ALA treatment alone induced expression of antioxidant genes (POD, APX and CAT), thus increased the activities of antioxidant enzymes (POD, APX and CAT) (Fig 4). Under Cd^2+^ stress, expression of these activities was also up-regulated with ALA treatment. The expression patterns of these genes were totally consistent with enzyme activities. The GR activity, which can be partially reflected by GSH/GSSG ratio [52], was significantly increased upon treatment with ALA under Cd^2+^ stress condition in the present study. These increased activities as well as enhanced gene expression may be beneficial to ASA-GSH cycle. Similarly, Nishihara et al. [53] suggested that higher GR activity triggered by ALA in spinach led to a large pool of GSH, which could make the ASA-GSH cycle more efficient. Zhang et al. [31] found that the activity of SOD of oilseed rape leaves with ALA and herbicide ZJ0273 was significantly higher over the control. Nishihara et al. [53] also demonstrated a slightly higher SOD activity during foliar application of ALA at 0.6 and 1.8 mM to the spinach leaves than that of control. These present findings support this view; however, particular mechanisms require further investigation.

### Proteomic features of *B*. *napus* leaves under application of ALA and Cd

#### CO_2_ assimilation / photosynthesis

Heavy metals can adversely affect the photosynthetic pathway [19] by depressing proteins involved in carbon fixation such as Rubisco proteins. These proteins directly control the photosynthetic mechanism of green plants. Their degradation and/or fragmentation in metal tolerant and non-tolerant plants are due to redox (e.g. copper and cadmium) and non-redox (e.g. mercury, cobalt, manganese, zinc) heavy metals [54, 55]. In the present study, we found one down-regulated (A06) and one up-regulated (B21) RuBisCO protein under Cd^2+^ stress alone conditions. The intensity of the up-regulated one was 1.75, while that of down-regulated one was 2.51 as compared to control (Table 1 and 2). So, the down-regulation was more, which conveyed the idea that the Calvin cycle was slowed down. The inhibition of this protein may be due to reduced photosynthetic efficiency and chlorophyll pigments of the *B*. *napus* leaves in Cd^2+^ stressed conditions [16]. Furthermore, Kieffer et al. [54] showed that proteins involved in carbon fixation and photosynthesis were repressed in young poplar leaves due to Cd^2+^ treatment. The down-regulation of RuBisCO and other photosynthesis- related enzymes have also been observed in response to metals other than Cd^2+^ [56]. Moreover, one up-regulated RuBisCO protein (F17) was also found with the application of ALA under Cd^2+^ stress (Table 2). The intensity of this up-regulated protein was 1.81 as compared to Cd^2+^ stress conditions. The up-regulation of RuBisCO with the application of ALA may be due to that ALA is a precursor for the biosynthesis of tetrapyrrols, and it can improve the photosynthetic efficiency of the *B*. *napus* leaves under Cd^2+^ stress [16]. Additionally, the alleviation of Cd^2+^induced of photosynthesis-related proteins by ALA supports the findings of Nwugo et al. [20], who concluded that Si could alleviate the Cd^2+^induced photosynthesis-related proteins in rice.

#### Protein synthesis and regulation

In present study, proteins were found to be involved in the synthesis and regulation of Ribonucleo protein (Table 1, spot no. A18) 30S ribosomal protein (Table 1, spot no. A24 and Table 2, spot F19) and Regulator of ribonuclease (Table 2, spot no. B45). Results showed that two proteins (spot id A18, A24) were down-regulated (A18, A24) and one protein (spot id B45) was up-regulated under Cd^2+^ stress (Table 1 and 2). These findings revealed the fact that ALA alleviated Cd^2+^induced toxicity effects on regulation/protein synthesis-related proteins. Similar to present findings, Basile et al. [57] stated that Cd^2+^ repressed PTPase at the transcriptional level in the liverwort. Further, Durand et al. [58] also described that Cd^2+^ repressed Elongation factor 1-γ and S-adenosylmethionine synthetase in poplar cambial proteome. Moreover, results also depicted that one up-regulated protein (F19) was found with the application of ALA under the Cd^2+^ stress (Table 2). Its intensity increased several fold over the relevant protein in Cd^2+^ stressed alone conditions. The fact that exogenously applied ALA alleviated the Cd^2+^toxicity effects on the above-mentioned protein synthesis/regulation-related proteins in *B*. *napus* plants is interesting.

#### Carbohydrate metabolism

In this study, we observed Cd^2+^ induced up-regulation of epithiospecifier protein (Table 2, spot no. B11) especially in the absence of ALA. It is well documented that epithiospecifier protein promotes the hydrolysis of glucosinolates to nitriles [54]. This protein commonly presents in plants that containing glucosinolates. Glucosinolates without terminal alkene functions may also be converted to nitriles under various conditions [59]. Kieffer et al. [54] also suggested that Cd^2+^ stress could limit the carbon availability by slowing down of photosynthetic carbon fixation, which can cause the plants to induce processes involved in carbohydrate catabolism [60]. However, an up-regulated protein (Dihydrolipoyl dehydrogenase 1, F09) of this category was found with the application of ALA under Cd^2+^ stress conditions (Table 2). The intensity of protein was 1.85 fold higher as compared to Cd^2+^ stress alone conditions. The up-regulation of this carbohydrate metabolism protein might be due to that ALA improved the photosynthetic carbon fixation process [45] and ultimately, carbon availability increased in the plant leaves.

#### Stress related proteins

In this experiment, two stress related proteins were found under Cd^2+^ and/or ALA i.e. one was down-regulated and one was up-regulated. The down-regulated protein (E20) was “thioredoxin-like protein” and found under the combine treatment of ALA and Cd^2+^ stress (Table 1). The stress related protein was suppressed under the combine treatment of ALA and Cd^2+^, which showed that plants did not go under stress conditions at this level [9]. However, “Thioredoxin-like protein” acts as an electron donor, which can catalyze the reduction of cysteine disulfides in proteins. Thus, in the cytoplasm, thioredoxins have a role in the catalytic cycle of redox enzymes, such as ribonucleotide reductase, and they also play role in preventing protein disulfide bridge formation in the cytoplasm [61]. Moreover, up-regulated stress related protein (B39) was found in the Cd^2+^ stress alone condition, which confirm the possibility that plant goes under stress at this level [22]. Further, up-regulation in stress related proteins was also observed in soybean roots under Cd^2+^ stress [54].

#### Redox homeostasis, defense and transport proteins

Proteins involved in redox homeostasis are usually involved in the prevention of oxidative stress, which is induced by reactive oxygen species (ROS). ROS are byproducts of electron transport and redox reactions from metabolic processes such as photosynthesis and respiration. The production of ROS has been shown to be markedly increased under conditions of abiotic stress [62]. Cd^2+^ toxicity has been shown to induce the production of ROS, most likely indirectly because the redox potential of Cd^2+^ is too low to participate in Fenton-like redox reactions [63]. However, the effect of Cd^2+^ on antioxidants or proteins involved in redox homeostasis is complex and appears to be dependent on growth conditions, species, and level/duration of Cd^2+^ exposure. However, superoxide dismutase (SOD) acts as a primary defense against ROS by converting O_2_^-^ to O_2_ and H_2_O_2_, which requires a specific metal cofactor [64]. In the present study, we found one up-regulated redox homeostasis protein (B47), one up-regulated defense related protein (B28) and one up-regulated transport protein (B53) under Cd^2+^ stress, in the absence of ALA. These results confirmed that under Cd stress, plants gone under stress conditions and that’s why, these proteins were up-regulated to accumulate the stress [1]. However, there appeared no protein under the combine treatment of ALA and Cd^2+^, which confirmed the findings of Nwugo et al. [9], who stated that exogenously applied Si alleviated Cd^2+^ induced stress in plants. Similarly, Kieffer et al. [54] also found an up-regulation in redox homeostasis related proteins in popular under Cd^2+^ stress.

#### *B*. *napus* leaf proteins regulated by ALA and Cd

Data showed only three proteins regarding this category and all were down-regulated. These proteins were “2, 3-bisphosphoglycerate-independent phosphoglycerate mutase 1” (C03), Proteasome subunit alpha type-1-A (C14) and “Thiamine thiazole synthase” (C15) and these proteins contained different function categories such as carbohydrate metabolism, dehydration of damaged proteins and catalysis, respectively (Table 1). It is considered that protein “2, 3-bisphosphoglycerate-independent phosphoglycerate mutase” is a metallo enzyme found particularly in archaea and some eubacteria. It is responsible for the interconversion of 2-phosphoglycerate and 3-phosphoglycerate [65]. However, proteasome contains multicatalytic proteinase complex which is characterized by its ability to cleave peptides with Arg, Phe, Tyr, Leu, and Glu adjacent to the leaving group at neutral or slightly basic pH [66]. Moreover, “thiamine thiazole synthase” catalyzes the conversion of NAD and glycine to adenosine diphosphate 5-(2-hydroxyethyl)-4-methylthiazole-2-carboxylic acid (ADT), an adenylated thiazole intermediate. The enzyme can only undergo a single turnover, which suggests it is a suicide enzyme. It may have additional roles in adaptation to various stress conditions and in DNA damage tolerance [67]. Additionally, present findings confirmed that these three proteins were down-regulated under the combine treatment of ALA and Cd^2+^ as compared to control. It might be suggested that there was lower stress and ultimately, less number of damaged proteins and catalytic process was found at this level.

## Conclusions

The present study provides initial evidence showing that the role of ALA in plants might not be limited to that of a mechanical role but that ALA might be actively involved in the regulation of biochemical processes particularly the modulation of protein production. Generally, results from this investigation provide insights into gene expressions and post-transcriptional regulatory mechanisms induced by ALA in plants under abiotic stress conditions. Further studies to determine the effect of ALA-induced stress tolerance in *B*. *napus*, the sequential influence of ALA on gene/protein expression especially during periods of stress, and possible ALA-protein interactions are currently being explored.

## Supporting Information

S1 Fig Down-regulated protein spots from the corresponding 2-DE gels in the leaves of *Brassica napus* cv. ZS 758 under 500 μM Cd^2+^ alone (with A lettering) as compared to control; under the combine treatment of ALA and 500 μM Cd^2+^ (with E lettering) as compared to Cd^2+^ alone and under the combine treatment of ALA and 500 μM Cd^2+^ (with C lettering) as compared to control conditions. (TIF)

S2 Fig Up-regulated protein spots from the corresponding 2-DE gels in the leaves of *Brassica napus* cv. ZS 758 under 500 μM Cd^2+^ alone (with B lettering) as compared to control; under the combine treatment of ALA and 500 μM Cd^2+^ (with F lettering) as compared to Cd^2+^ alone and under the combine treatment of ALA and 500 μM Cd^2+^ (with D lettering) as compared to control conditions. (TIF)
