# Supplementary figures and images for: Regulation of Cadmium-Induced Proteomic and Metabolic Changes by 5-Aminolevulinic Acid in Leaves of Brassica napus L
Source: PLoS One. 2015 Apr 24;10(4):e0123328. doi: 10.1371/journal.pone.0123328 (PMC4409391; doi:10.1371/journal.pone.0123328)

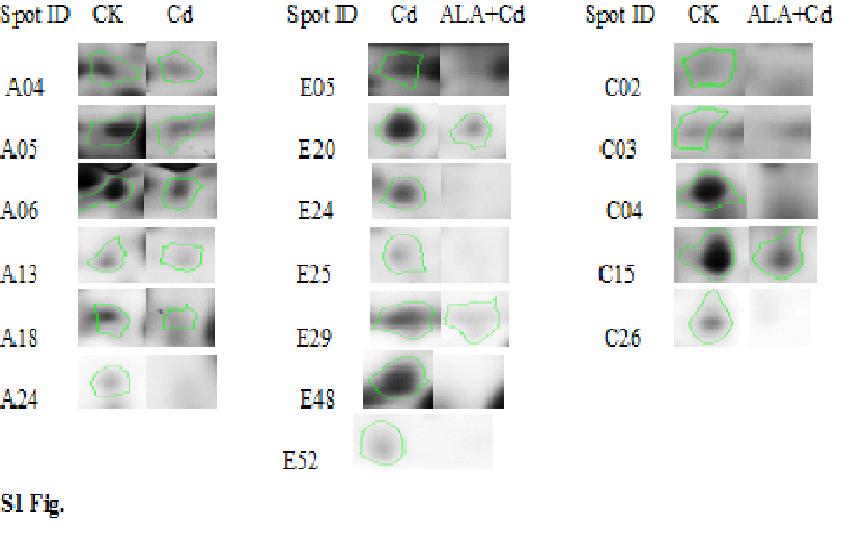

Supplement: S1 Fig — (TIF) [file pone.0123328.s001.tif]

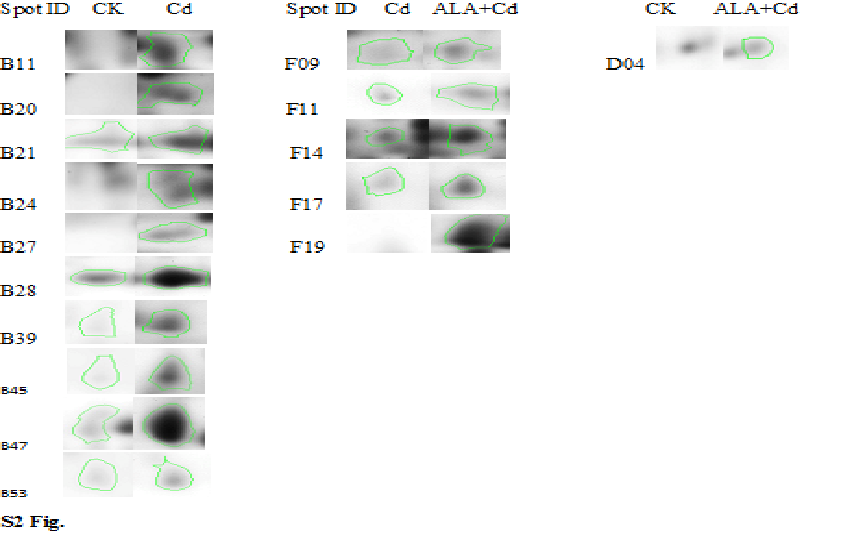

Supplement: S2 Fig — (TIF) [file pone.0123328.s002.tif]
